# Supplementary figures and images for: Effect of Sprouting on Biomolecular and Antioxidant Features of Common Buckwheat (Fagopyrum esculentum)
Source: Foods. 2023 May 18;12(10):2047. doi: 10.3390/foods12102047 (PMC10217231; doi:10.3390/foods12102047)

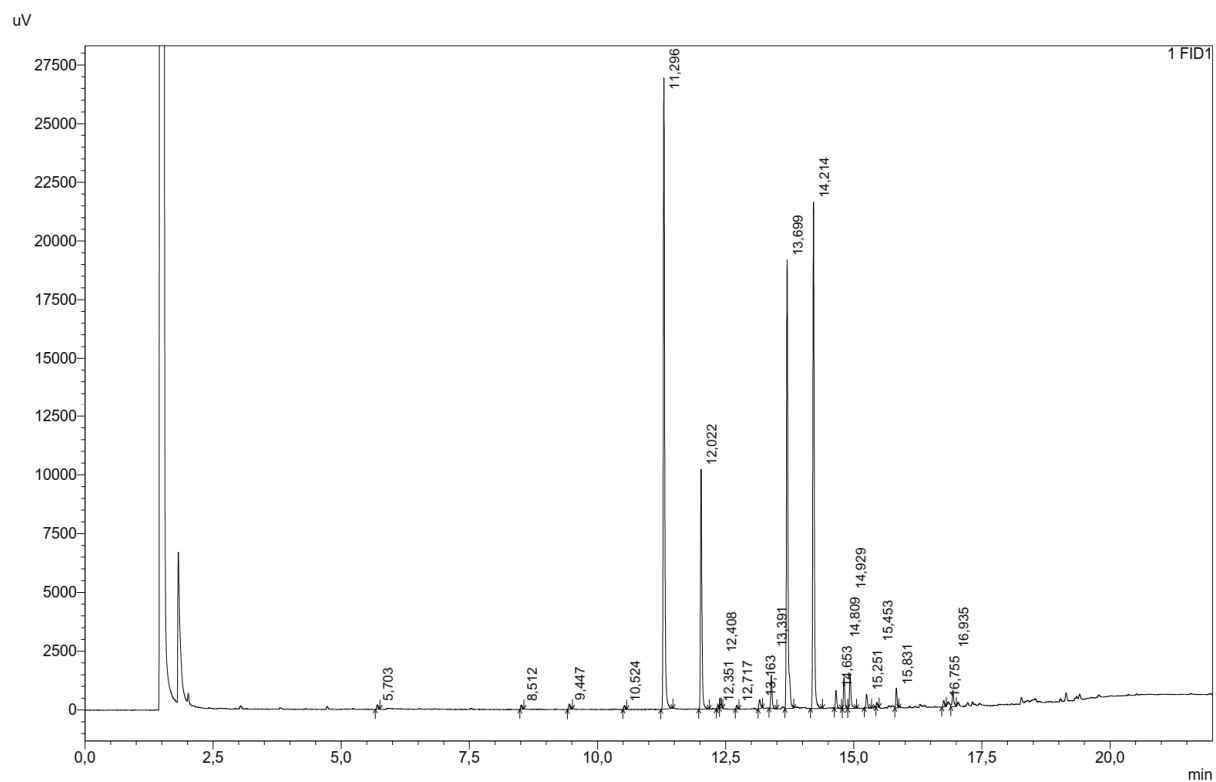

**Figure S1.** Representative chromatogram of BW-0.

Supplement: Supplementary file 1 [file foods-12-02047-s001.zip › foods-2388761-supplementary.pdf]
